# Assessment of Fe, Cr, and Zn in New Commercial Invasive Fish Species and their Public Health Implications

**DOI:** 10.1007/s12011-026-05121-6

**Published:** 2026-04-29

**Authors:** Duygu Ceren Çağlan Kaya, Deniz İnnal

**Affiliations:** Department of Biology, The Faculty of Arts and Sciences, Burdur Mehmet Akif Ersoy University, Burdur, 15100 Türkiye

**Keywords:** Health risk assessment, Antalya Bay, Mediterranean Sea, Bioaccumulation, PTEs

## Abstract

PTE contamination is widely recognized as a serious threat to both environmental and human health. In the Mediterranean Sea, three recently introduced non-native fish species (*Nemipterus randalli*,* Saurida lessepsianus*, and *Siganus rivulatus*) have successfully established self-sustaining populations following their introduction. While their ecological impacts remain incompletely understood, these invasive species have entered local markets and are increasingly consumed, giving them economic value. This study aimed to evaluate the concentrations of iron (Fe), chromium (Cr), and zinc (Zn) and assess potential health risks associated with their consumption. Specimens were collected during both the dry and wet seasons, and PTE concentrations in muscle tissue were determined by inductively coupled plasma optical emission spectrometry (ICP-OES). Chromium (Cr) levels were significantly higher in the wet season compared to the dry season (*p* < 0.05). Mean Zn and Fe concentrations were within national and international permissible limits, whereas Cr contamination may pose a concern for consumers, particularly in *S. rivulatus*. Risk assessment based on Estimated Daily Intake (EDI), Target Hazard Quotients (THQ), and Total THQ (TTHQ) was conducted for all three fish species included in the study. However, potential health risks were identified only for *S. lessepsianus* and *S. rivulatus*, in which TTHQ values for Fe exceeded the safety threshold of 1.

## Introduction

Potentially toxic elements (PTEs) are recognised as hazardous pollutants due to their toxicity and persistence in the environment. PTE contamination in aquatic organisms, mainly caused by human activities like agricultural runoff, industrial discharges, and household wastewater, poses a major threat to environmental and public health [1, 2]. These persistent contaminants are notable for their toxicity, bioaccumulative capacity, and resistance to natural degradation, altering biological functions and disrupting ecosystem dynamics in both freshwater and marine environments. PTEs enter the food web through direct uptake or trophic transfer, leading to biomagnification at higher levels [3–7].

Fish are an essential component of the human diet, providing high-quality proteins, essential minerals, vitamins, and unsaturated fatty acids [8, 9]. However, their nutritional value is compromised by such pollution. Exposure induces structural and functional damage in tissues, posing a dual threat to fish health and human consumers [10, 11]. Consequently, monitoring PTE concentrations and assessing the health risks to humans who consume contaminated seafood remain global research priorities [12, 13].

Species such as *N. randalli*,* S. lessepsianus*, and *S. rivulatus* are among the most prominent Lessepsian migrant fish species that have successfully formed self-sustaining populations in the Eastern Mediterranean following the opening of the Suez Canal. These species, originally from the Red Sea and the Indo-Pacific region, have adapted well to their new environment and now represent a considerable component of the region’s marine biodiversity [14, 15]. Although their precise ecological impacts are still under investigation, their growing abundance suggests they play an increasingly important role in local marine food webs.

*Nemipterus randalli* (Randall’s threadfin bream), a demersal species from the family Nemipteridae, inhabits sandy and muddy substrates at moderate depths. It exhibits carnivorous feeding habits, preying on benthic invertebrates and small fish (Its rapid population growth, combined with its commercial value, has increased its relevance to local fisheries in the Levantine Basin [16, 17]. *Saurida lessepsianus*, a lizardfish belonging to the family Synodontidae, is a benthic predator frequently encountered in soft-bottom habitats. It primarily feeds on small teleosts and crustaceans. Due to its abundance and active predatory behavior, it has become an ecologically significant species in Eastern Mediterranean ecosystems [18, 19]. *Siganus rivulatus*, commonly known as the marbled spinefoot, is an herbivorous species in the family Siganidae. It feeds extensively on macroalgae and seagrass, often contributing to shifts in benthic community structure, especially in shallow coastal habitats [20]. Its proliferation has raised concerns regarding overgrazing and the subsequent loss of native algal cover [21].

The establishment and expansion of these species exemplify the complex ecological consequences of Lessepsian migration. This situation highlights the necessity for continuous monitoring, ecological impact assessments, and adaptive management strategies to ensure the ecological balance and sustainable use of Eastern Mediterranean marine resources [22].

The Antalya Bay, located in the eastern Mediterranean, is a distinctive marine ecosystem that has been the focus of numerous studies on heavy metal contamination and its impact on biodiversity and environmental health. Previous investigations have reported varying concentrations of elements such as Cu, Zn, Pb, Cd, Cr, Fe, Mn, Ni, and Co in different species inhabiting the area [23–27]. Also, health risk assessments, involving EDI and THQ calculations, have reported on whether trace element concentrations in edible tissues remain within or exceed national and international safety thresholds [28–30].

Global seafood consumption has increased markedly over the past six decades, driven by shifting consumer preferences, technological advancements, and rising incomes. In 2019, global seafood consumption reached 157 million tonnes, with 72% consumed in Asia. The leading countries in seafood consumption are China, Indonesia, India, the United States, and Japan. On a global scale, fish accounted for 17% of animal protein intake in 2019 and represented 7% of total protein consumption [31]. Per capita fish consumption worldwide rose from 9 kg in 1961 to 20.2 kg in 2020. In 2019, fish accounted for 75% of seafood consumption, mollusks 12%, and crustaceans 13%. Income levels and dietary culture largely influence variations in consumption among countries. In 2019, low-income, food-deficient countries reported an average per capita fish consumption of 5.4 kg, compared to 15.2 kg in middle-income countries and 26.5 kg in high-income countries [31]. Globally, total seafood production reached approximately 178 million tonnes in 2020, of which 157 million tonnes were used directly for food supply, while the remaining 20 million tonnes were directed to non-food purposes such as fishmeal and fish oil production [31]. Given the increasing role of seafood in global diets and trade, monitoring its safety has become a priority. PTE contamination in fish represents a significant public health concern due to its toxicity, persistence, and bioaccumulative potential. Such contaminants can enter aquatic food webs through anthropogenic inputs and eventually pose health risks to consumers.

This study is especially relevant with three recently introduced non-native fish species in the Eastern Mediterranean — *N. randalli*,* S. lessepsianus*, and *S. rivulatus*. Originally from the Red Sea and the Indo-Pacific, these species have successfully formed self-sustaining populations following Lessepsian migration, are sold in local markets, and are regularly consumed by coastal communities. The present study aims to determine the concentrations of selected PTEs in the edible tissues of these species and to assess potential health risks associated with their consumption, thereby contributing to current knowledge and supporting sustainable seafood safety management in the region.

## Materials and Methods

### The Study Area

**Fig. 1:**
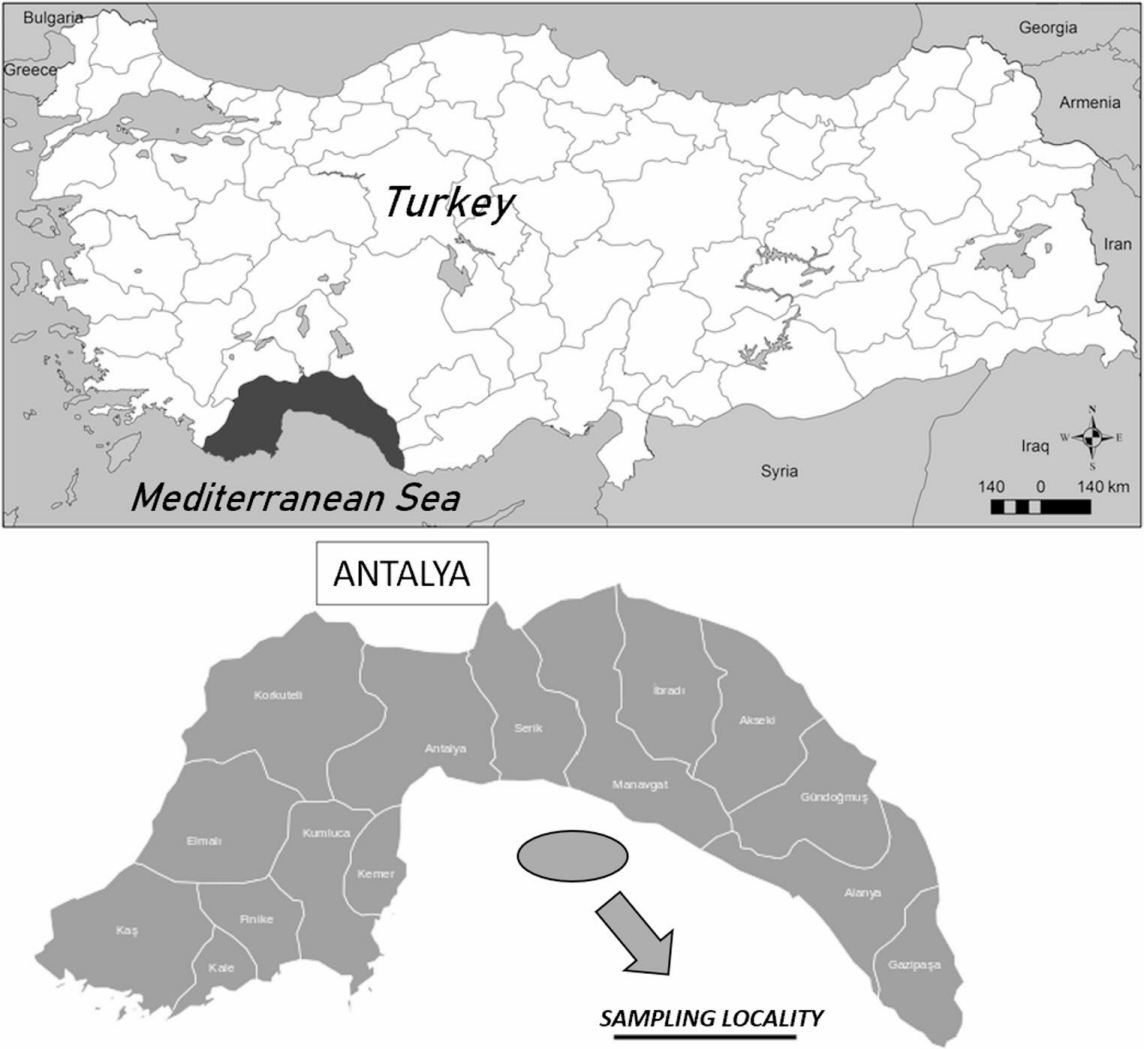
Sampling locality at Antalya Bay

Antalya Bay, located in the northeastern Mediterranean along the southern coast of Turkey, is the largest and most oligotrophic area and is recognized for its significant ecological diversity and environmental importance [32]. It shows a distinct fish community structure compared to other regions along the Turkish Mediterranean coast and the Turkish seas. This semi-enclosed coastal marine ecosystem exhibits a variety of ecological features. The gulf hosts diverse marine life, including endemic and migratory species, coral habitats, and a wide variety of benthic and pelagic organisms [20, 33]. Antalya, located in the Mediterranean basin of Türkiye and a leading destination for tourism revenue, is an important city where more than 2.5 million people live and are visited by over 15 million tourists each year. The region’s popularity as a tourism hotspot results in coastal urbanization, hotel construction, and beach modifications, leading to habitat loss and fragmentation [34]. Marina and port expansions also alter the hydrodynamics of the coastal zone, which affects sediment transport and local ecosystems. Figure 1 illustrates the geographical location of the study area.

It is known that global warming, urbanization, and extensive land loss are becoming increasingly inevitable for Mediterranean countries, with climate change impacts being projected with greater precision and severity [35]. Due to its semi-enclosed nature, the Antalya Bay is highly sensitive to marine pollution, particularly from terrestrial and anthropogenic runoff. Water circulation is limited, which can exacerbate eutrophication caused by agricultural runoff and urban wastewater [36].

The marine ecosystem of Antalya Bay faces significant ecological pressures, primarily driven by coastal development, intensive tourism, overfishing, and the proliferation of invasive species. A critical threat among these is contamination originating from anthropogenic activities. The influx of untreated or partially treated sewage, industrial effluents, and agricultural runoff introduces excessive nutrients, heavy metals and microplastics into the basin [37]. These pollutants can trigger eutrophication and harmful algal blooms, leading to a decline in water quality that endangers sensitive habitats, such as *Posidonia oceanica* meadows [38].

Furthermore, as part of the Eastern Mediterranean ‘Lessepsian migration’ corridor, Antalya Bay faces a continuous influx of Red Sea species via the Suez Canal. Invasive species such as *N. randalli*, *S. lessepsianus*, and *S. rivulatus* have altered native community structures and compete with endemic species for habitat and food [16, 39, 40]. These invasions are further intensified by rising sea surface temperatures and acidification, which promote the expansion and establishment of non-native tropical species within the basin [41].

### Sampling Procedure

A total of 60 samples (10 of each season) were obtained via local fishermen from Antalya Bay in two seasons (2022 dry and 2024 rainy) and then transported to the laboratory in cold conditions.

### Chemical Analyses

Fish samples were washed with distilled water, then the total fish length was measured in millimeters (mm) and the weight in grams (g) before dissection. Accurately 0.5 g (wet weight) muscle tissue sample from each individual was weighed in TFM tubes. 9mL nitric acid (HNO_3_ 65%, Sigma Aldrich, St. Louis, MO, USA) and 1 mL hydrogen peroxide (H_2_O_2_ 30%, Sigma Aldrich) were added to each tube. The samples were digested with a microwave digestion system (Milestone Start D, Milestone Srl, Sorisole, Bergamo, Italy) using a two-stage temperature program (in the first stage, the microwave combustion system was increased to 110 °C for 15 min, and in the second stage, it was held at 110 °C for 15 min). Inductively coupled plasma-optic emission spectroscopy (ICP-OES, Perkin Elmer Optima 8000, Waltham, MA, USA) was used to analyze digested samples for concentrations of studied elements. ICP-OES operation conditions are/were power of 1450 W, axial plasma view, sampling depth 10 mm, nebulizer gas flow of 0.55 L/min, and flow rate 1.50 µL/min. Analytical quality confirmed by analyses of certified reference material (TUBITAK, National Metrology Institute, UME EnvCRM 03, Gebze, Kocaeli, TÜRKİYE). The recovery values for Fe, Cr, and Zn were 76.9%, 94.29%, and 95.0%, respectively.

The measured concentrations were compared with the maximum residue limits established by international organizations. Regulatory limits established by the U.S. Environmental Protection Agency (USEPA), European Commission (EC), European Food Safety Authority (EFSA), Food and Agriculture Organization (FAO), U.S. Food and Drug Administration (FDA), Turkish Food Codex (TFC), the Joint FAO/WHO Expert Committee on Food Additives (JECFA), and the World Health Organization (WHO) were considered.

### Health Risk Analyses

The health risks associated with the consumption of PTE-contaminated fishery products were assessed based on EDI, THQ, and TTHQ analysis methods.

#### Estimated Daily Intake (EDI)

The estimated daily intake for each PTE was calculated using the formula below.

1 $$EDI=\;MC\left(\mu g/g\right)\times IRd\;\left(g\right)/\;BWa\;\left(kg\right)$$

MC represents the mean element concentration of an element in fish tissue (µg/g), while IRd is daily fish ingestion rate (g/day) and BWa indicates adult body weight (kg) [42]. In health risk assessments, BWa is evaluated commonly assumed to be 70 kg for adults [43]. In Türkiye, the per capita fish consumption rate has been reported as 6.14 kg/year (16. 8 g/day) [44].

#### Target Hazard Quotient (THQ)

Non-carcinogenic health risks associated with PTE intake through fish consumption were assessed using the target hazard quotient (THQ). This approach provides an effective means of estimating the potential human health risks arising from exposure to these elements [45]. PTEs that accumulate in tissue were evaluated using the THQ [28, 29]. THQ represents the ratio of elemental exposure to humans from food sources against the threshold level where no adverse effects are anticipated. A calculated THQ value of 1 or below means that the accumulation level of the relevant element in food does not pose a risk for human consumption [46]. The target hazard quotient (THQ) was calculated using Eq. (2), as recommended by the U.S. Environmental Protection Agency [47]:

2 $$THQ=\left[\left(EF\times ED\times IRd\times MC\right)/\left(RfD\times BWa\times ATn\right)\right]\times10^{-3}$$

where EF denotes exposure frequency (365 days year⁻¹), ED is the exposure duration, assumed to be the average life expectancy of 26 years (US EPA, 2023), and IRd represents the daily fish consumption rate, estimated as 6.14 kg/year (16.8 g/day) for Turkish households (GDFA, [44]). MC refers to the concentration of PTEs in edible fish tissues (mg kg⁻¹). BWa represents the average adult body weight, taken as 70 kg, while ATn is the average exposure time for non-carcinogenic effects, calculated as 365 days × 26 years [48, 49]. RfD denotes the oral reference dose (mg kg⁻¹ day⁻¹) for each element, which was set at 0.001 for Fe, 0.003 for Cr, and 0.3 for Zn.

## Result and Discussion

PTEs analyses of fish muscle tissue were performed obtained from a total of 60 individuals of *N. randalli*,* S. lessepsianus and S. rivulatus.* Concentrations of Fe, Cr, and Zn in the muscles are presented in Table 1. Limit values determined by various organizations were also shown. The mean PTE concentrations in the muscle tissue of *N. randalli* were found to be 2.11 mg/kg for Fe, 1.91 mg/kg for Cr, and 3.25 mg/kg for Zn. In *S. lessepsianus*, the respective concentrations were 6.74 mg/kg for Fe, 2.96 mg/kg for Cr, and 5.25 mg/kg for Zn, while *S. rivulatus* exhibited mean concentrations of 5.70 mg/kg for Fe, 3.15 mg/kg for Cr, and 8.38 mg/kg for Zn.

**Table 1:** Fe, Cr, and Zn concentration in the muscle tissue (mg/kg ww)

Species	Fe (mg/kg ww)	Cr (mg/kg ww)	Zn (mg/kg ww)
N. randalli*	3.79	0.43	3.62
N. randalli**	0.42	3.38	2.88
S. lessepsianus*	13.33	0.23	1.93
S. lessepsianus**	0.15	5.69	8.57
S. rivulatus*	11.29	0.17	13.15
S. rivulatus**	0.11	6.12	3.61
Limit values			
FAO [50, 51]	100	0.01	30
WHO [52]	-	0.15	40
EU [53, 54])	-	0.01	-
TFC [55]	50	-	-

*2022 dry season ** 2024 rainy season

A comparative assessment of these values indicates interspecific variability in element accumulation. Specifically, *S. lessepsianus* exhibited the highest Fe content, whereas *S. rivulatus* showed elevated levels of both Cr and Zn. In contrast, *N. randalli* consistently exhibited the lowest concentrations across all examined elements. These findings suggest species-specific differences in bioaccumulation capacity, which may be influenced by ecological factors such as feeding habits, habitat preferences, and trophic position.

The paired t-test showed that only chromium (Cr) showed a statistically significant seasonal difference between the 2022 dry and 2024 rainy seasons, with higher values detected in the rainy season (*p* < 0.05). Although iron (Fe) concentrations were higher during the dry season, this difference was not statistically significant (*p* = 0.091). Zinc (Zn) levels did not differ between seasons (*p* = 0.820). Zinc (Zn) concentrations showed no seasonal difference (*p* = 0.820). Overall, these findings indicate that statistically detectable seasonal variation was limited to Cr under the present sampling conditions, whereas Fe and Zn did not show significant seasonal variations.

Concentrations detected in muscle tissues were compared to international food safety standards; the detected levels of Zn in all three species remained below the maximum permissible limit established by the FAO [51]. It was observed that, except for Cr, the concentrations in all species remained below the established limit values in both seasons. Similarly, Fe concentrations were lower than the recommended dietary upper limit for fish muscle, indicating no immediate risk from Fe intake. However, Cr concentrations approached or, in the case of *S. rivulatus* (3.154 mg/kg), exceeded the FAO/WHO guideline limits for fish muscle, suggesting a potential health risk associated with frequent consumption of this species. These findings highlight that while Zn and Fe levels are within safe limits, Cr contamination may pose a concern for human consumers, particularly in species such as *S. rivulatus*.

The consumption of fish from areas affected by untreated industrial, agricultural, and domestic discharges significantly increases human dietary exposure to environmental contaminants.

### Iron

Iron is an essential trace element involved in physiological functions, and both deficiency and excessive dietary intake may pose health risks like anemia, cardiovascular, neurological, hepatic, and renal impairments [56–58] reported that oral RfD of iron is 0.7 µg/kg day^− 1^. According to Table 1, Iron (Fe) was consistently higher during the dry season (3.79–13.33 mg/kg ww) compared to the rainy season (0.11–0.42 mg/kg ww).

In the present study, Fe concentrations in *N. randalli* muscle were 3.79 mg/kg ww (2022 dry season) and 0.42 mg/kg ww (2024 rainy season). These values are considerably lower than those reported in Mersin Bay (80.77 ± 5.16 µg/g ww) [59] and İskenderun Bay (105.9 µg/g dw) [60]. Seasonal differences were observed, with Fe levels declining during the rainy season. Compared to previously published regional data, Fe levels in the present study were relatively low (Table 2). In *S. lessepsianus* Fe concentrations in muscle were 13.33 mg/kg ww (2022 dry season) and 0.15 mg/kg ww (2024 rainy season). These values are lower than those reported from İskenderun and Mersin Bays, where concentrations frequently exceeded 50 µg/g [60, 61]. In *S. rivulatus*, Fe concentrations differed between the two sampling periods: 11.29 mg/kg ww in the 2022 dry season and only 0.11 mg/kg ww in the 2024 rainy season. Compared to earlier reports from Antalya Bay, Fe concentrations in 2022 (11.29 mg/kg ww) were lower than reported by Ateş et al., [62] (39.1 ± 23.4 mg/kg ww) but higher than of Can et al., [28] (5.47 ± 2.00 mg/kg ww). The 2024 rainy season concentration was considerably lower than values reported in earlier studies [59, 60, 63], suggesting temporal variability or species-specific differences in Fe accumulation (Table 2).

**Table 2:** Comparison of Fe, Cr, and Zn concentrations in the muscle tissues of invasive fish species (*N. randalli*,* S. lessepsianus*,* S. rivulatus*) reported from various regions across the Mediterranean Sea

	Fe	Cr	Zn	Locality	Ref.
*N. randalli*	3.79 (mg/kg ww) *	0.43 (mg/kg ww) *	3.62 (mg/kg ww) *	Antalya Bay	Present Study
	0.42 (mg/kg ww) **	3.38 (mg/kg ww) **	2.88 (mg/kg ww) **	Antalya Bay	Present Study
	80.77 ± 5.16 (µg/g).	-	16.11 ± 0.53 (µg/g).	Mersin Bay	Külcü et al., [59]
	+ 1 age: 27.6 + 2 age :41.7 + 3 age: 53.3 (µg/g dw)	ND	+ 1 age: 8.4 + 2 age: 10.3 + 3 age: 9.2 (µg/g dw)	Mersin Bay	Göçmen et al., [64]
	Min 16.60 ± 1.63 (spring) Max 38.89 ± 14.41 (autumn) (µg/g dw)	min 0.32 ± 0.02 (winter) max 0.71 ± 0.19 (autumn) (µg/g dw)	Min 11.50 ± 0.65 (autumn) Max 16.19 ± 2.12 (spring) (µg/g dw)	Taşucu Region	Karaytuğ et al., [65]
	105,9 (µg/g dw)	-	13.6 (µg/g dw)	İskenderun Bay	Çiftçi et al., [60]
	44.8 (µg/g dw)	-	13.5 (µg/g dw)	Mersin Bay	Çiftçi et al., [60]
	BDL	BDL	1.76 ± 0.92 (mg/kg ww)	Antalya	Korkmaz et al., [30]
	-	0.31 ± 0.003 (µg/g ww)	-	Mediterranean Sea, Egypt,	Hasanein et al., [66]
*S. lessepsianus*	13.33 (mg/kg ww)*	0.23 (mg/kg ww)*	1.93 (mg/kg ww)*	Antalya Bay	Present Study
	0.15 (mg/kg ww)**	5.69 (mg/kg ww)**	8.57 (mg/kg ww)**	Antalya Bay	Present Study
	Max 92.77 ± 17.91 (spring) Min 11.83 ± 2.26 (fall) (mg/g dw)	-	Max 18.91 ± 1.78 (winter) Min 2.45 ± 0.40 (fall) (mg/g dw)	Iskenderun Bay	Manaşırlı et al., [63]
	Min 17.26 ± 2.33 (spring) Max 41.92 ± 11.71 (autumn) (µg/g dw)	Min 0.33 ± 0.03 (winter) Max 0.94 ± 0.17 (autumn) (µg/g dw)	Min 14.48 ± 1.45 (winter) Max 20.16 ± 2.69 (autumn) (µg/g dw)	Taşucu Region	Karaytuğ et al., [65]
	Min 17.86 ± 0.53 (autumn) Max 38.27 ± 2.15 (spring) (mg/100 g)	Min 1.66 ± 0.01 (summer) Max 3.16 ± 0.01 (spring) (mg/100 g)	Max 40.96 ± 0.43 (winter) Min 17.86 ± 0.53 (spring) (mg/100 g)	Mersin Bay	Kosker, [61]
	61.8 (µg/g dw)	-	14.3 (µg/g dw)	İskenderun bay	Çiftçi et al., [60]
	73.1 (µg/g dw)	-	15.9 (µg/g dw)	Mersin bay	Çiftçi et al., [60]
	BDL	BDL	4.50 ± 1.83 (mg/kg ww)	Samandağ	Korkmaz et al., [30]
	BDL	BDL	3.48 ± 0.68 (mg/kg ww)	İskenderun	Korkmaz et al., [30]
	BDL	0.04 ± 0.06 (mg/kg ww)	3.13 ± 0.97 (mg/kg ww)	Silifke	Korkmaz et al., [30]
	-	-	19.2 (µg/g)	Coastline of Antalya province	Koraltan et al., [67]
	14.9 ± 2.5 (µg/g ww)	-	-	Egyptian Mediterranean Sea coastal area between Alexandria and Sidi Kerir	Hasanein et al., [66]
*S. rivulatus*	11.29 (mg/kg ww)*	0.17 (mg/kg ww)*	13.15 (mg/kg ww)*	Antalya Bay	Present Study
	0.11 (mg/kg ww)**	6.12 (mg/kg ww)**	3.61 (mg/kg ww)**	Antalya Bay	Present Study
	39.1 ±23.4 (mg/kg ww)	0.36 ± 0.05 (mg/kg ww)	5.36 ± 0.55 (mg/kg ww)	Antalya Bay	Ateş et al., [62]
	5.47 ± 2.00 (µg/g ww)	0.20 ± 0.15 (µg/g ww)	3.34 ± 0.65 (µg/g ww)	Antalya Bay	Can et al., [28]

*2022 dry season ** 2024 rainy season

### Chromium

Chromium (Cr) is present in seawater primarily as trivalent (Cr³⁺) and hexavalent (Cr⁶⁺) species, which differ in their environmental behavior and biological relevance [68–70].

According to Table 1, Cr concentrations exhibited seasonal variation, with values of 0.43 mg/kg ww during the dry season and 3.38 mg/kg ww in the rainy season. During the dry season, Cr concentrations were relatively uniform across species (0.17–0.43 mg/kg ww). In contrast, during the rainy season, concentrations increased, with the highest values detected in *S. rivulatus* (6.12 mg/kg ww) and *S. lessepsianus* (5.69 mg/kg ww), followed by *N. randalli* (3.38 mg/kg ww). For *N. randalli*, Cr concentrations in the present study were higher than those reported for Mersin and İskenderun Bays (< 1 µg/g dw), indicating spatial variability among eastern Mediterranean regions. In *S. lessepsianus*, Cr concentrations increased from 0.23 mg/kg ww (dry season) to 5.69 mg/kg ww (rainy season), and the latter value exceeded concentrations reported from Mersin and Taşucu Bays (< 1 µg/g dw). In *S. rivulatus*, Cr concentrations were 0.17 mg/kg ww in the dry season and 6.12 mg/kg ww in the rainy season. Previous records from Antalya Bay reported comparatively lower Cr concentrations (0.36 ± 0.05 mg/kg ww; [62]; 0.20 ± 0.15 mg/kg ww; [28]), indicating notable temporal variation across sampling years (Table 2).

### Zinc

Zinc (Zn) is an essential trace element involved in enzymatic and structural functions, serving as a cofactor for numerous metabolic processes [71]. The oral reference dose (RfD) for Zn has been reported as 300 µg kg⁻¹ day⁻¹ (USEPA, 2020). Adverse health effects, including neurodegenerative illnesses like Alzheimer’s disease, increased cancer risk, accelerated aging, diabetes, depression, and other significant neurological conditions including Wilson’s disease, may result from chronic ingestion that exceeds this threshold.

Highest Zn concentrations were observed in *S. rivulatus* during the dry season (13.15 mg/kg ww) (Table 1). *S. lessepsianus* exhibited the lowest Zn concentrations in the dry season (1.93 mg/kg ww), followed by a marked increase during the rainy season (8.57 mg/kg ww). *N. randalli* showed relatively stable concentrations of Zn (2.88–3.62 mg/kg ww) across seasons.

In *N. randalli*, Zn concentrations were 3.62 mg/kg ww (2022 dry season) and 2.88 mg/kg ww (2024 rainy season). These values were lower than those previously reported for Mersin Bay (16.11 ± 0.53 µg/g; [59]) and İskenderun Bay (13.6 µg/g; [60]). In *S. lessepsianus*, Zn concentrations were 1.93 mg/kg ww (dry season) and 8.57 mg/kg ww (rainy season), which were also lower than values reported for İskenderun Bay (14.3 µg/g dw) and Mersin Bay (15.9 µg/g dw) (Table 2).

### Risk analysis

Estimated Daily Intake values of Fe, Cr and Zn for a person through the consumption of fishes and also data of PTDI/PTWI and PTMI suggested by organizations are listed in Table 3.

**Table 3:** Estimated daily intake values of Fe, Cr and Zn average concentrations are determined in muscle tissue (µg/kg bw/day)

Species	EDI		
	Fe	Cr	Zn
*Nemipterus randalli*	0.51	0.05	0.78
*Saurida lessepsianus*	1.62	0.71	1.26
*Siganus rivulatus*	1.37	0.76	2.01
*PMTDI	800 µg/kg bw day ^a^		
*PTWI		0.7 µg/kg bw week ^b^	^c^7 µg/kg bw week ^c^
*PTMI			^d^ 25 µg/kg bw month ^d^

* PMTDI, PTWI, PTMI values for an adult human 70 kg bw

^a^FAO, [50], ^b^WHO, [72], ^cd^JECFA [73]

According to the data published in 2019 by the General Directorate of Fisheries and Aquaculture of the Ministry of Agriculture and Forestry of the Republic of Türkiye, the average annual fish consumption per capita in Türkiye is 6.14 kg. In Table 2, assuming a daily consumption of 16.8 g of fish meat by a 70 kg individual, the concentrations of elements that could be ingested were calculated. The obtained values were then compared with the elements’ PMTDI (Provisional Maximum Tolerable Daily Intake), PTWI (Provisional Tolerable Weekly Intake), and PTMI (Provisional Tolerable Monthly Intake) limits. As seen in Table 3, Cr concentrations in *Saurida lessepsianus* and *Siganus rivulatus* exceeded commonly reported reference limits. This pattern indicates that consumers of these species may experience higher dietary exposure to Cr. Zinc concentrations also surpassed the recommended daily intake values in the same species, whereas Fe concentrations remained below established safety thresholds.

The non-carcinogenic risk assessment based on Target Hazard Quotient (THQ) and Total Target Hazard Quotient (TTHQ) values provides important insights into the potential health implications of PTE intake through fish consumption. The results of the non-carcinogenic health risk assessments (THQ and TTHQ) for the three investigated species, along with comparative risk data from the literature, are summarized in Table 4. Based on the oral reference dose (RfD) values for the analyzed elements (Cr: 0.003; Zn: 0.3; Fe: 0.7 mg/kg/day), all calculated risk indices remained well below the critical threshold of 1, indicating no immediate health risk to consumers.

**Table 4:** THQ estimates from average Fe, Cr, and Zn concentrations in muscle tissue and comparison with earlier studies from Antalya Bay

Species	THQ			**TTHQ	Ref.
	Cr	Zn	Fe		
*RfDo	0.003	0.3	0.7		
*N.randalli*	0.152	0.003	0.505	0.66	This study
*S.lessepsianus*	0.24	0.004	*1.617*	*1.861*	This study
*S. rivulatus*	0.25	0.007	*1.368*	*1.625*	This study
*Mullus barbatus*	-	0.0062	-		Yipel &Yersan, [74]
*Mugil cephalus*	-	0.0084	-		Yipel & Yersan, [74]
*Mullus barbatus*	0.0001	0.0019	0.0017	0.0037	Can et al., [28]
*Solea solea*	0.0001	0.0014	0.0025	0.0040	Can et al., [28]
*S. rivulatus*	0.0001	0.0011	0.0009	0.0021	Can et al., [28]

*RfDo; RFD Oral reference dose; mg/kg/day

** TTHQ = THQ (Fe) + THQ (Cr) + THQ(Zn)

The Target Hazard Quotient (THQ) values for Pb, Cr, and Zn were below 1 for *N. randalli*, indicating that the estimated non-carcinogenic risk from exposure to each element through consumption of this species remains within acceptable limits. The total Target Hazard Quotient (TTHQ), representing the combined contribution of Cr, Zn, and Fe, was likewise below 1 for *N. randalli*, confirming that regular consumption does not pose a substantial health hazard (Table 4). In contrast, in both *S. lessepsianus* and *S. rivulatus*, THQ values for Fe exceeded 1, and the corresponding TTHQ values for these species were also greater than 1. These results suggest comparatively higher non-carcinogenic hazard estimates for these species under the assessed exposure conditions, particularly considering the essential yet potentially toxic nature of iron when consumed in excess [56, 75].

As seen in Table 4 previously reported introductions about Fe, Cr, and Zn accumulation in various localities for three species and other species from Antalya Bay. In comparison, previously reported values *for Mullus barbatus*,* Mugil cephalus*, and *Solea solea* [28, 74] show THQ and TTHQ levels well below 1, indicating safe consumption levels with respect to Cr, Zn, and Fe exposure.

The lack of adequate wastewater treatment facilities and the subsequent discharge of untreated effluents into marine environments remain critical concerns for biodiversity. Consuming fish from areas affected by such industrial, agricultural, and domestic discharges may significantly increase human exposure to environmental contaminants through the diet. The elevated trace element concentrations and the resulting health risk indices observed in this study may be linked to anthropogenic pressures on the coastal ecosystem. This may provide a plausible explanation for why certain species, such as *S. lessepsianus* and *S. rivulatus*, exhibit risk values exceeding safety thresholds, potentially leading to the non-carcinogenic health risks identified in our assessment. Therefore, monitoring the bioaccumulation of trace elements in commercially valuable species is essential for assessing and mitigating public health risks in the region.

## Conclusion

In conclusion, this study highlights clear differences in interspecific and seasonal variations in Fe, Cr, and Zn accumulation among invasive fish in Antalya Bay. The results show that while average concentrations of Zn and Fe mostly stay within international safety standards, chromium (Cr) levels exhibit significant seasonal changes, with notably higher levels during the rainy season (*p* < 0.05). Our risk assessment reveals that *N. randalli* remains safely within consumption limits, with all risk indices (THQ and TTHQ) below the critical threshold of 1. In contrast, *S. lessepsianus* and *S. rivulatus* exhibit higher bioaccumulation potential, particularly for Fe, resulting in THQ and TTHQ values above 1. These findings suggest that eating *S. lessepsianus* and *S. rivulatus* could pose potential non-carcinogenic health risks under high exposure scenarios. Therefore, while these species have economic value, their consumption should be monitored to protect public health in the Mediterranean region.

## Data Availability

All data generated and analyzed during this study, are available from the corresponding author upon reasonable request.
